# Supplementary figures and images for: Effect of obesity on the acute response to SARS-CoV-2 infection and development of post-acute sequelae of COVID-19 (PASC) in nonhuman primates
Source: PLoS Pathog. 2025 Jul 24;21(7):e1012988. doi: 10.1371/journal.ppat.1012988 (PMC12289017; doi:10.1371/journal.ppat.1012988)

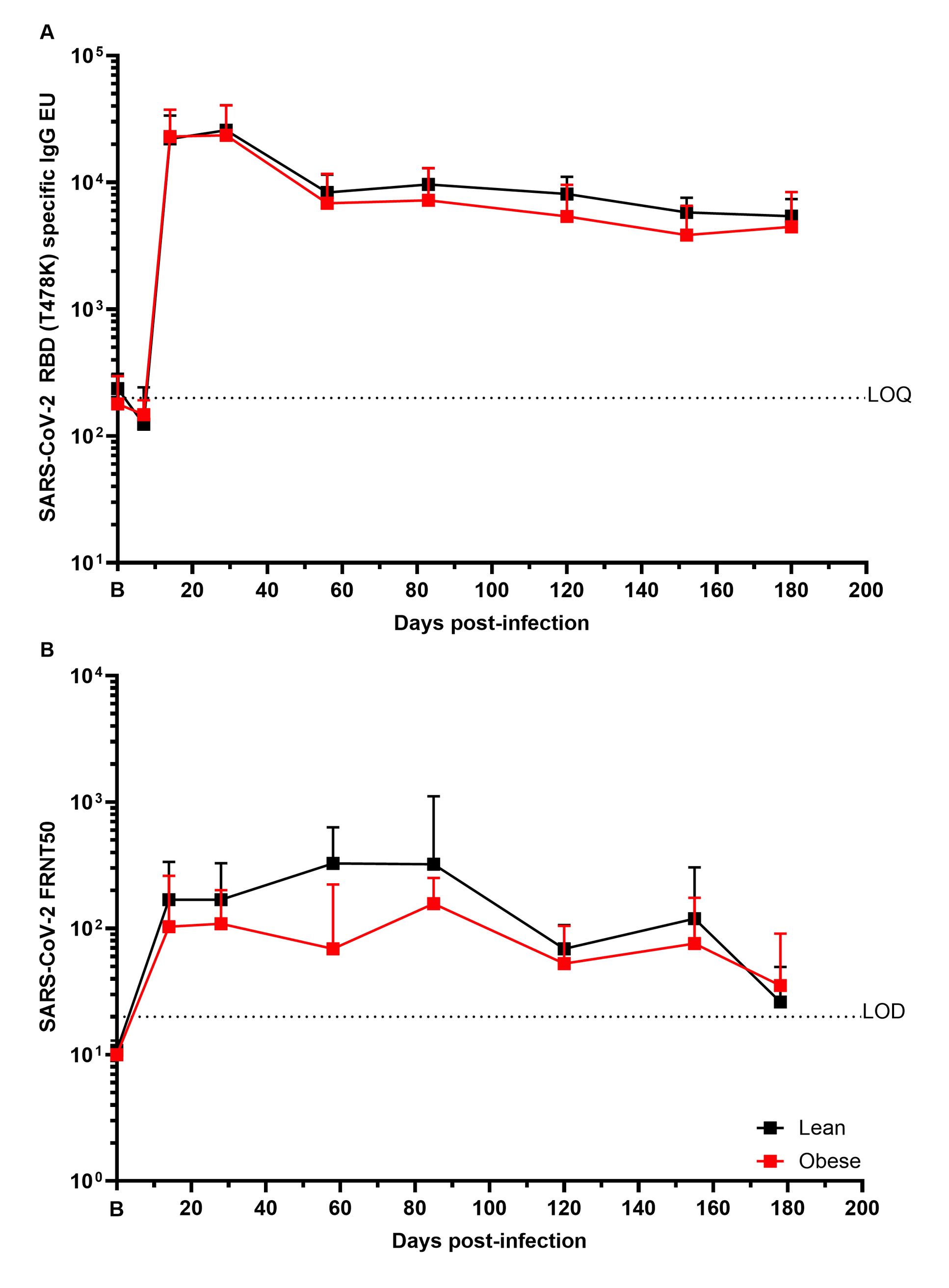

Supplement: S1 Fig — (A) RBD-binding IgG levels measured by ELISA. (B) SARS-CoV-2-specific neutralizing Ab levels. All data are GMT + /-95% CI. LOQ: limit of quantitation. LOD: Limit of detection. (TIF) [file ppat.1012988.s001.tif]

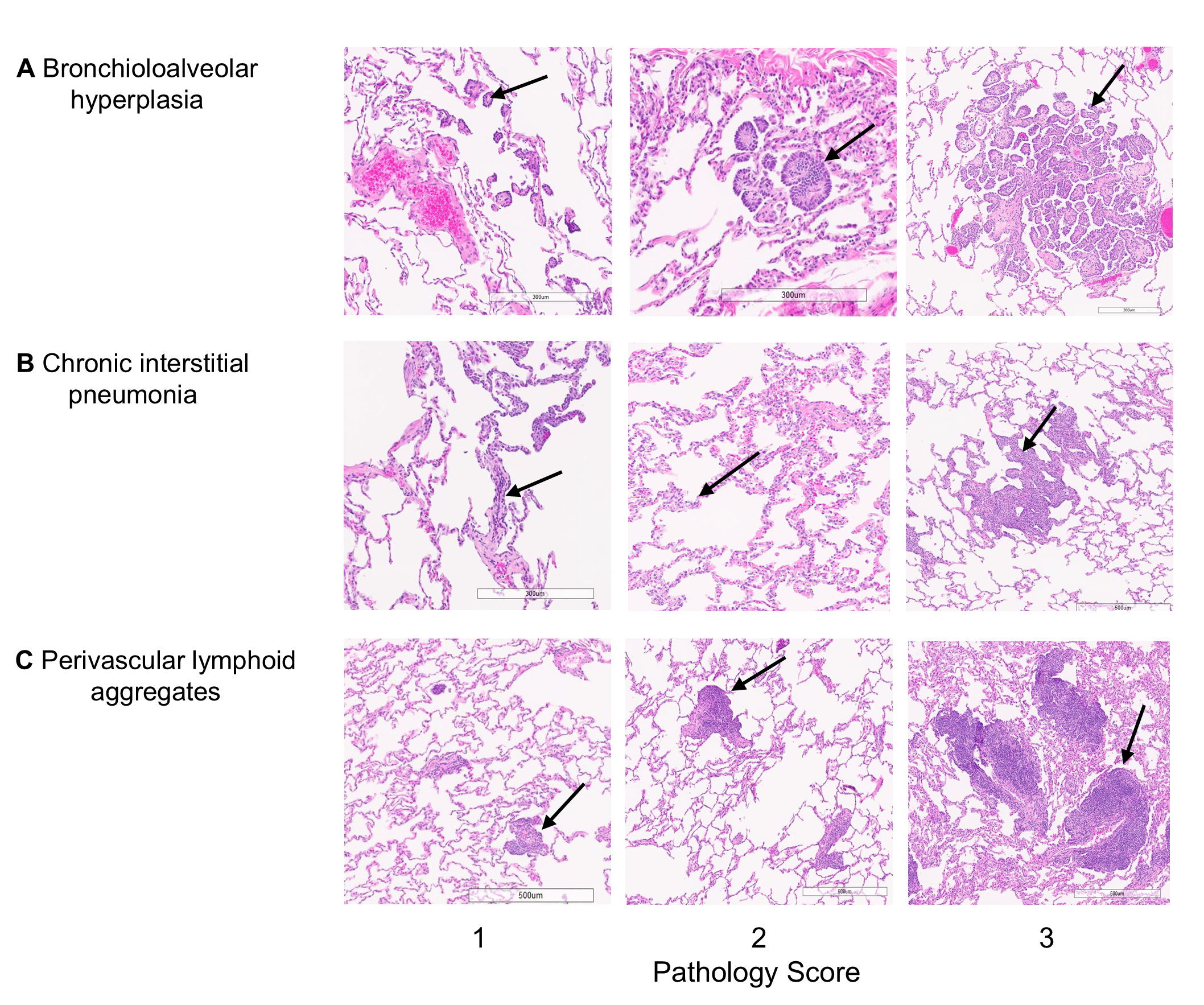

Supplement: S2 Fig — Scores range from 0 (no lesion, not pictured) up to a score of 3. (A) Top row, bronchioloalveolar hyperplasia (arrows) is characterized by clusters of hyperplastic epithelial cells arising from the terminal bronchiole, alveolar duct and alveolus. (B) Middle row, alveolar septa expanded with lymphocytes (arrows) are a feature of the chronic interstitial pneumonia observed here. (C) Bottom row, variable numbers of lymphocytes surround multiple blood vessels (arrows). The extent of pathology featured here ranges from minimal to mild to moderate, corresponding to scores of 1, 2 or 3. (TIF) [file ppat.1012988.s002.tif]

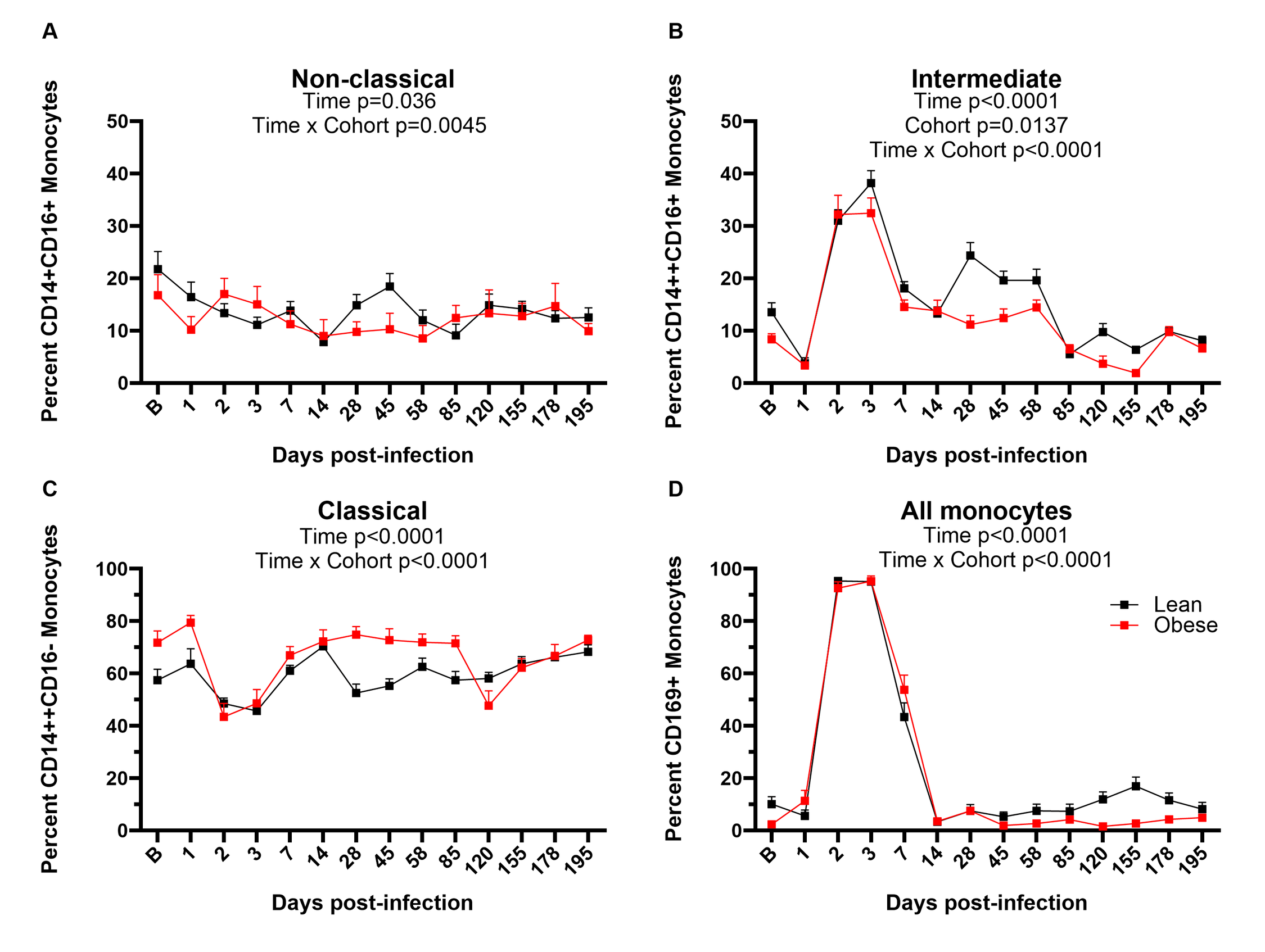

Supplement: S3 Fig — PBMCs were analyzed by flow cytometry to distinguish non-classical (A), intermediate (B), and classical (C) subpopulations, as well as the CD169 + population (D) using the antibodies and gating strategies shown in S2 Table and S7 Fig. All data are means ± SEM. Significance determined using mixed-effect analysis with Dunnett’s post-hoc for multiple comparisons test. (TIF) [file ppat.1012988.s003.tif]

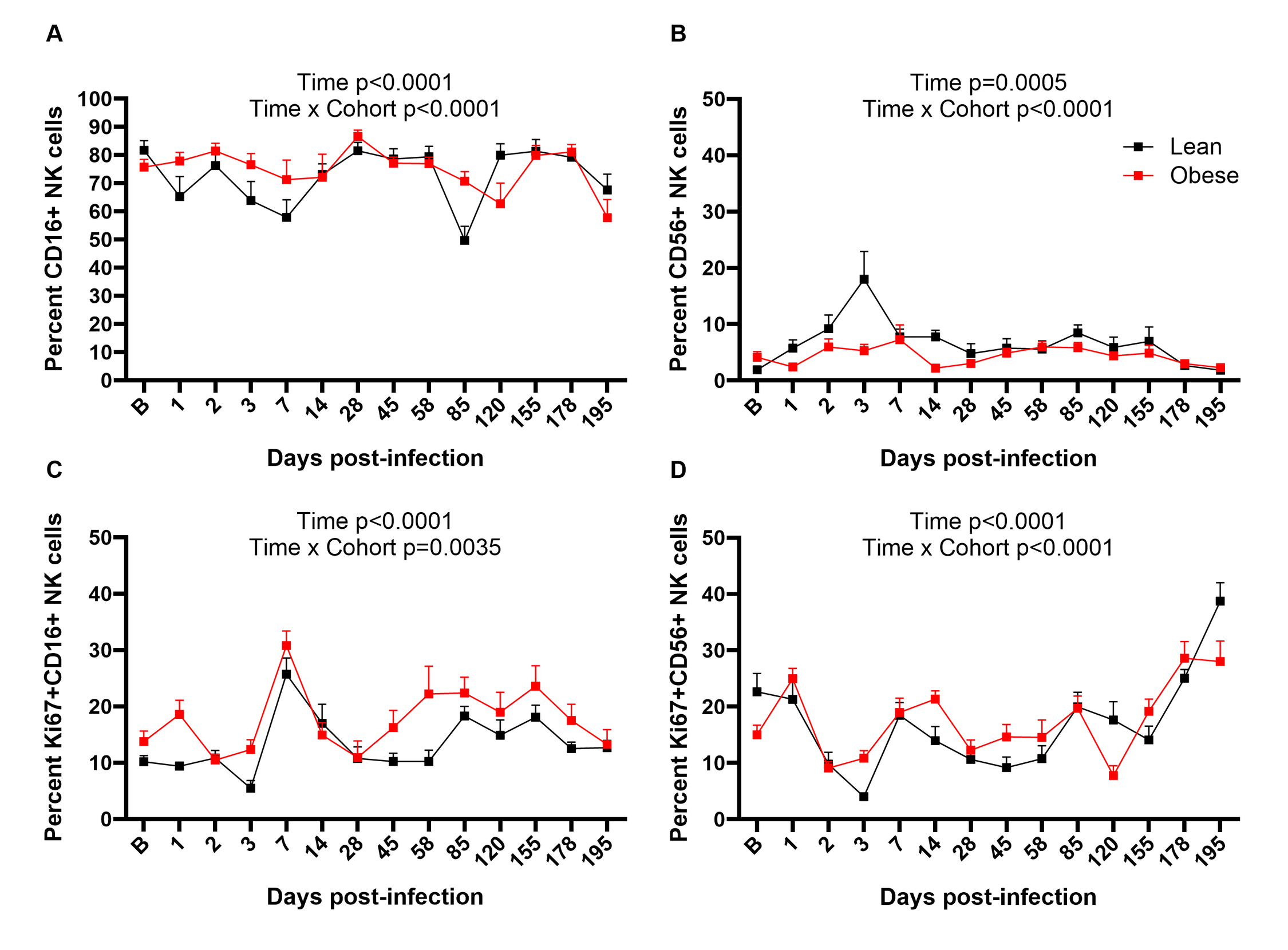

Supplement: S4 Fig — PBMCs were analyzed by flow cytometry to distinguish non-classical (A), intermediate (B), and classical (C) NK cell populations, as well as CD169 + NK cells (D) using antibodies and gating strategies shown in S2 Table and S7 Fig. All data are means ± SEM. Significance determined using mixed-effect analysis with Dunnett’s post-hoc for multiple comparisons test. (TIF) [file ppat.1012988.s004.tif]

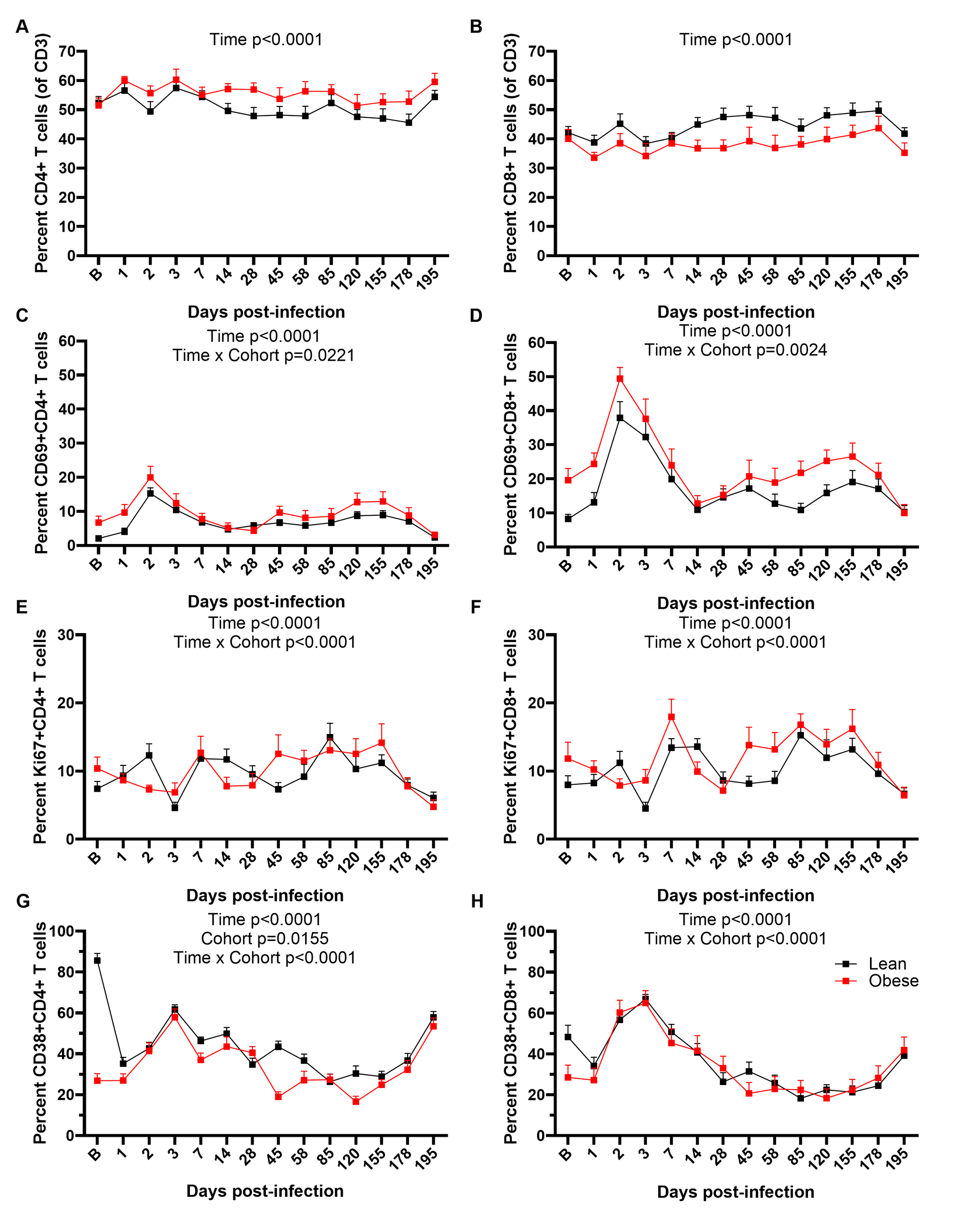

Supplement: S5 Fig — PBMCs were analyzed by flow cytometry to distinguish CD4+ (A), CD8+ (B), CD69 + CD4+ (C), CD69 + CD8+ (D), Ki67 + CD4+ (E), Ki67 + CD8+ (F), CD38 + CD4+ (G), and CD38 + CD8+ (H) T cells using antibodies and gating strategies shown in S2 Table and S7 Fig. All data are means ± SEM. Significance determined using mixed-effect analysis with Dunnett’s post-hoc for multiple comparisons test. (TIF) [file ppat.1012988.s005.tif]

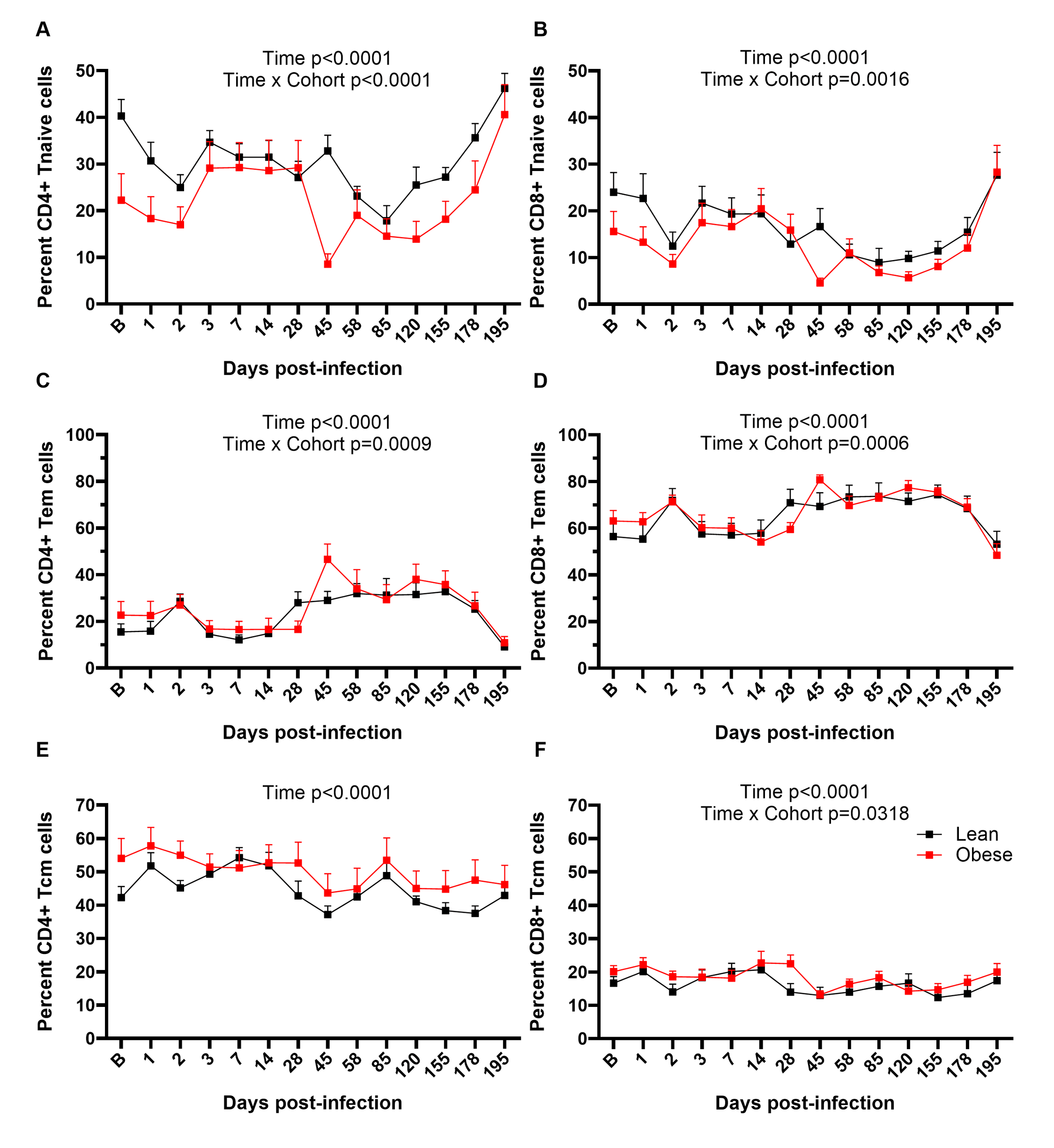

Supplement: S6 Fig — PBMCs were analyzed by flow cytometry to distinguish CD4 + naive (A), CD8 + naive (B), CD4 + effector memory (Tem) (C), CD8 + Tem (D), CD4+ central memory (Tcm) (E), and CD8 + Tcm (F) T cells using antibodies and gating strategies shown in S2 Table and S7Fig. All data are means ± SEM. Significance determined using mixed-effect analysis with Dunnett’s post-hoc for multiple comparisons test. (TIF) [file ppat.1012988.s006.tif]

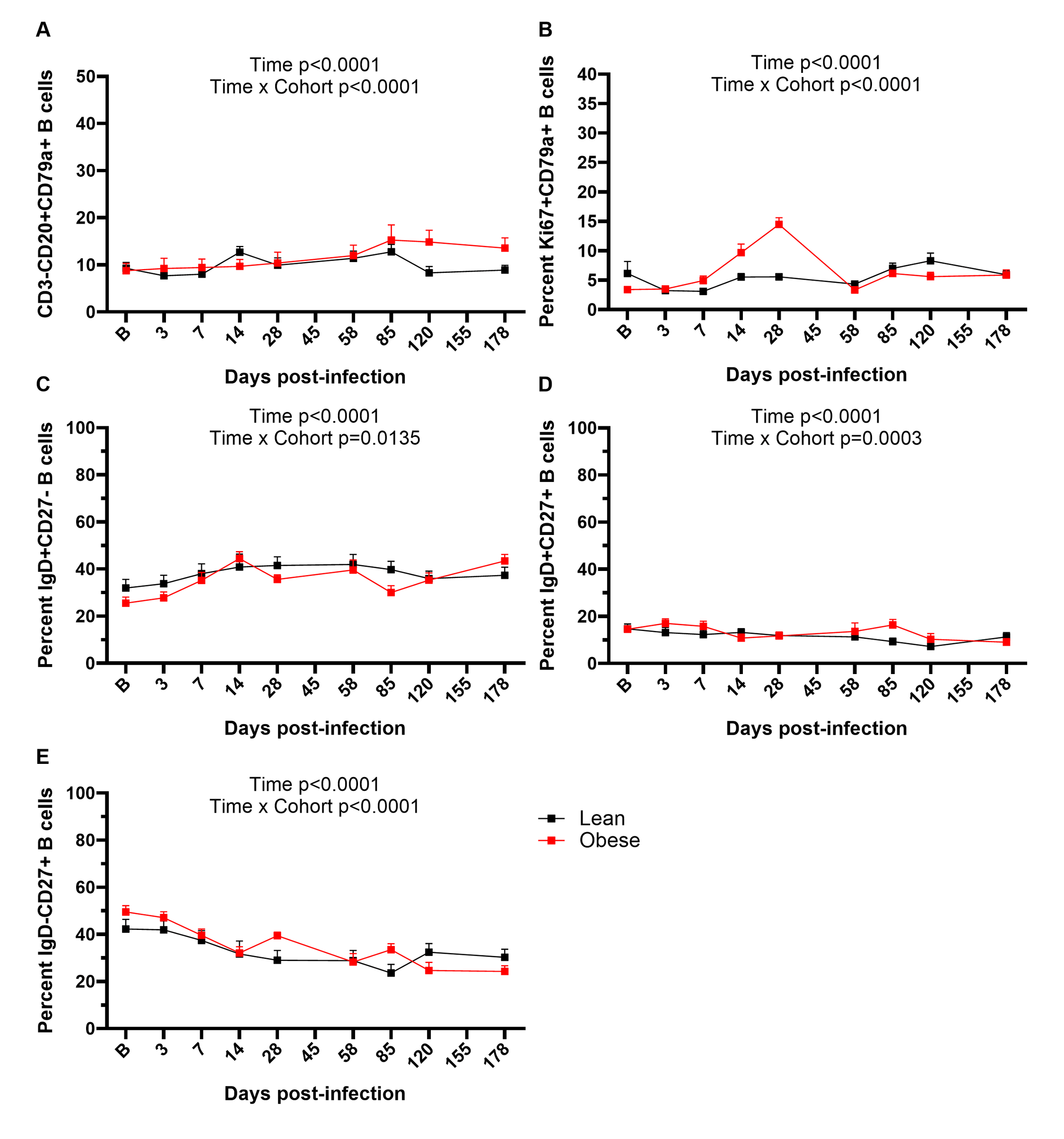

Supplement: S7 Fig — PBMCs were analyzed by flow cytometry to distinguish total CD3-CD20 + CD79a+ (A), activated Ki67 + CD3-CD20 + CD79a+ (B), naïve IgD + CD27- (C), IgM memory IgD + CD27+ (D), and class-switched memory IgD-CD27+ (E) B cells using antibodies and gating strategies shown in S2l Tableand S7 Fig. All data are means ± SEM. Significance determined using mixed-effect analysis with Dunnett’s post-hoc for multiple comparisons test. (TIF) [file ppat.1012988.s007.tif]

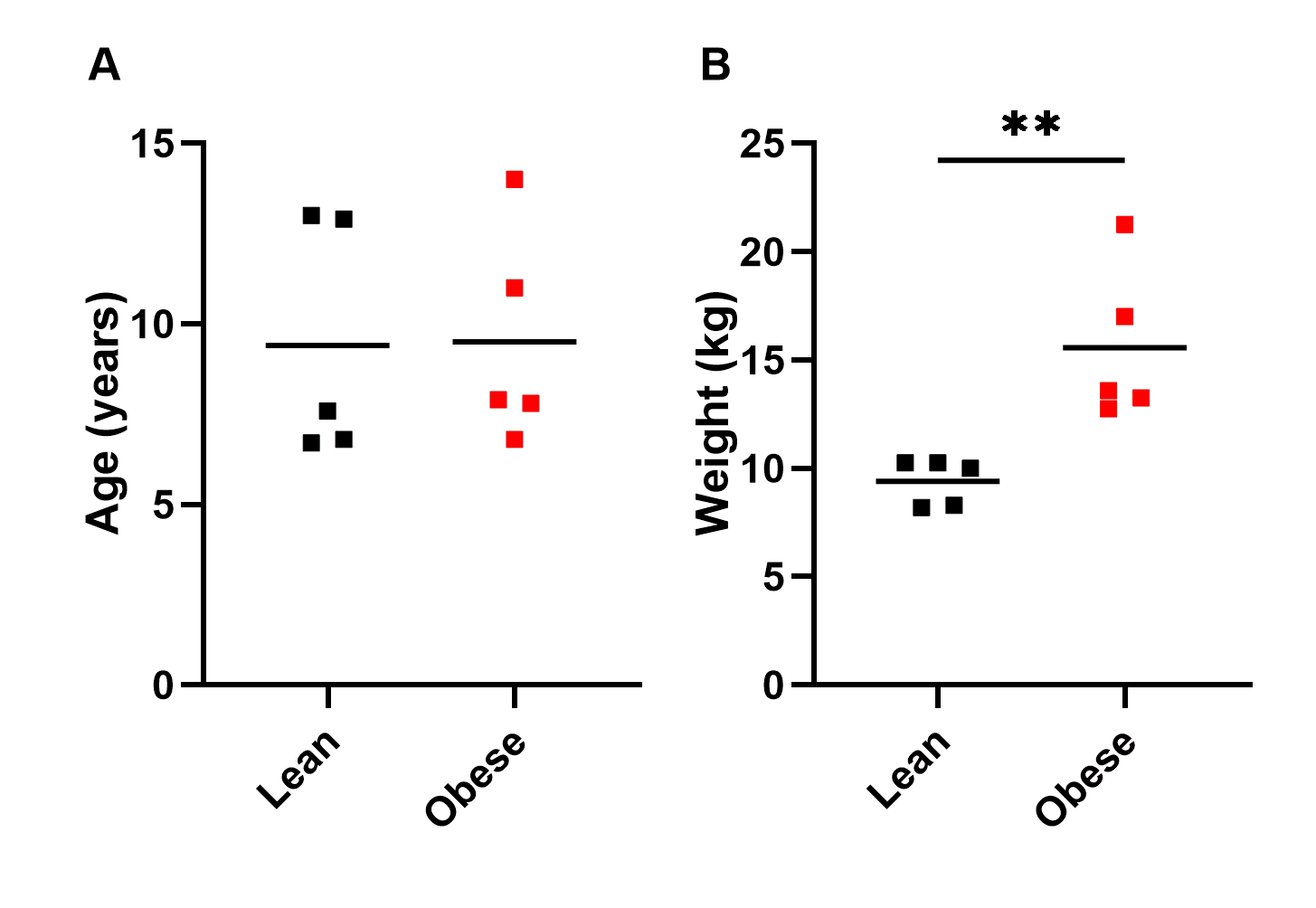

Supplement: S8 Fig — Significance was determined by unpaired 2-tailed t test. **, p < 0.01. (TIF) [file ppat.1012988.s008.tif]

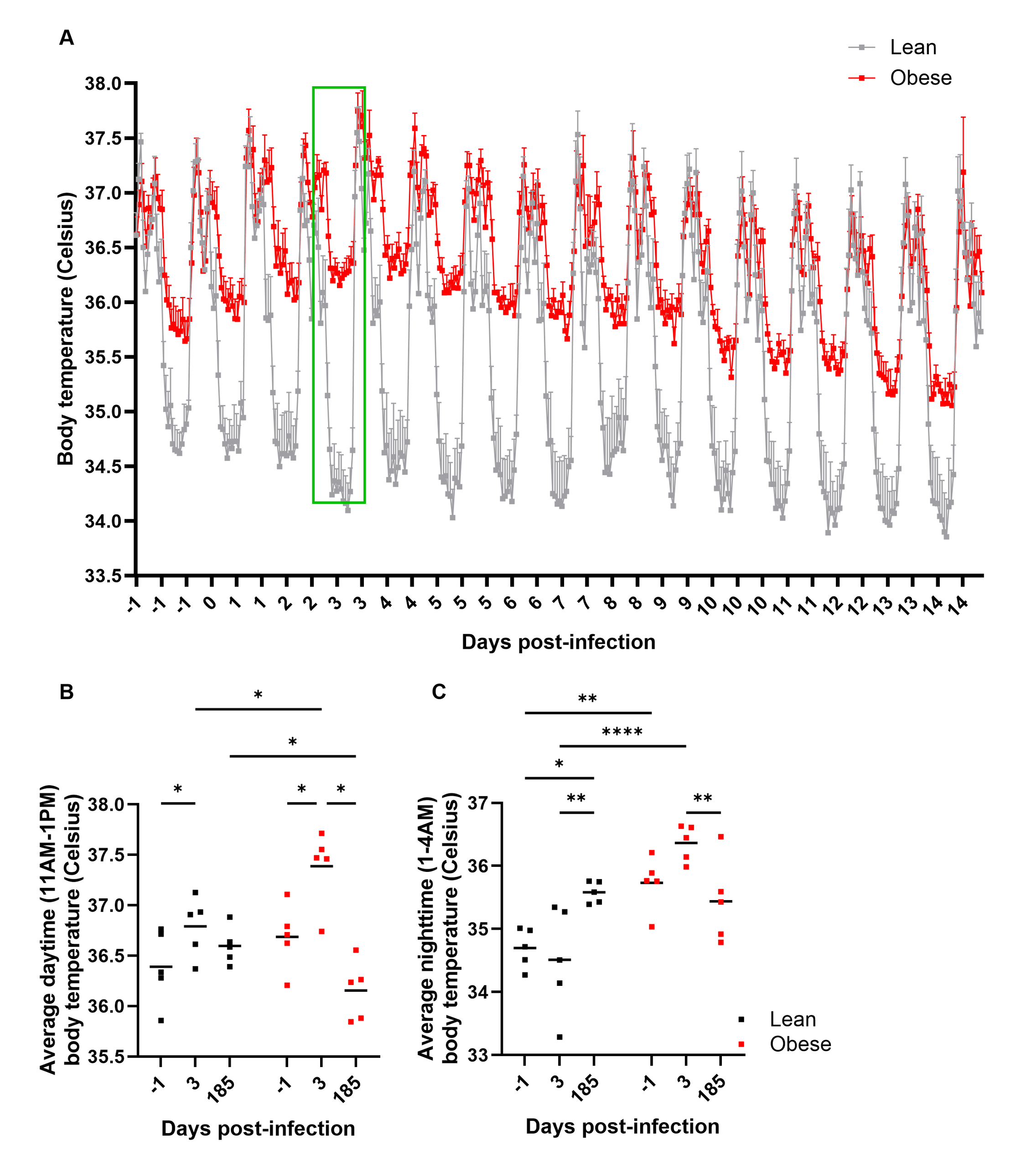

Supplement: S9 Fig — A. Circadian variation in BT in lean and obese animals from days -1–14 PI. Data are means ± SEM. B. Average daytime (11 AM-1 PM) and (C) nighttime (1–4 AM) BT at days -1, 3, and 185 PI. Significance determined by 2-way ANOVA w/Tukey’s multiple comparison test. *, p < 0.05; **, p < 0.01; ****, p < 0.0001. (TIF) [file ppat.1012988.s009.tif]

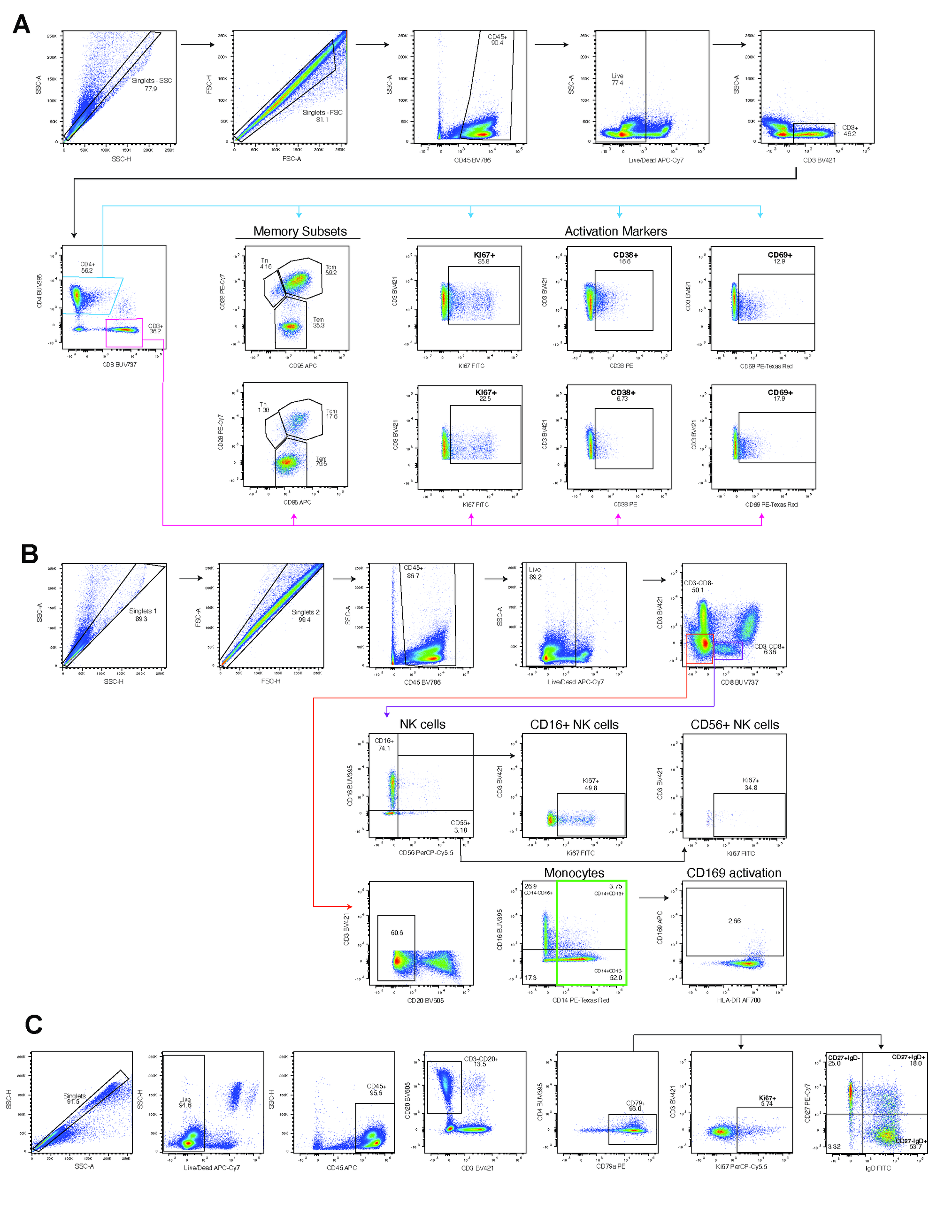

Supplement: S10 Fig — PBMCs were characterized for T cells (A), monocytes and NK cells (B), and B cells (C). (TIF) [file ppat.1012988.s010.tif]
